# Exploring patterns of substance use among highly vulnerable Black women at-risk for HIV through a syndemics framework: A qualitative study

**DOI:** 10.1371/journal.pone.0236247

**Published:** 2020-07-28

**Authors:** Liesl A. Nydegger, Kasey R. Claborn

**Affiliations:** 1 Department of Kinesiology and Health Education, College of Education, The University of Texas at Austin, Austin, TX, United States of America; 2 Department of Psychiatry, Dell Medical School, The University of Texas at Austin, Austin, TX, United States of America; San Diego State University, UNITED STATES

## Abstract

Black women who use alcohol, marijuana, and other drugs are disproportionately affected by health disparities. Black women’s HIV diagnosis rates are 15 times higher than White women, and is among the leading causes of death among Black women in the US. Previous studies support the association between substance use and HIV risk, yet it is essential to better understand the specific factors experienced within the context of substance misuse and recovery among vulnerable Black women at-risk for substance misuse, HIV, and adverse life experiences. We conducted qualitative interviews with 31 black women (age *M* = 32.13, range 18–57) four times over six months. Eligible participants were 18+ years, identified as a Black/African-American woman, had unprotected vaginal or anal sex with a man in the past 30 days, and spoke fluent English. All transcripts were transcribed verbatim and were analyzed used thematic content analysis. Two groups of participants emerged: 1) those in recovery from their drug of choice (n = 11, 7 of whom misused alcohol or marijuana during the study), and 2) those who misused their drug of choice during the study (active use group; n = 20). Four themes emerged in the context of substance use: cultural factors, structural factors (i.e., housing and employment), past and present adverse life experiences, and individual factors (i.e., substance use to cope with stress, self-medicating with substances for mental health symptoms, intimate partner violence, and sex exchange). While participants in both groups used substances to cope with regard to these factors, the recovery group tended to use substances at lower frequencies and did not relapse with their drug of choice during the study. The active use group reported more substance use with regard to structural factors and recent adverse life events, had more difficulty regarding employment, and less instances of intimate partner violence (IPV) but were more likely to cope using substances. Substance use interventions tailored to vulnerable Black women should consider including trauma-informed interventions and support groups that address the structural, social, and individual factors to better serve their needs.

## Introduction

Despite efforts to reduce health disparities across the United States, Black women continue to be disproportionately affected by both physical and mental health concerns including cancer, cardiovascular disease, HIV, depression, and substance use issues [1,2]. This population experiences concomitant economic, systemic challenges including discrimination and familial conflict barriers contributing to increased morbidity and mortality [3–5]. Consequently, increased rates of sex work, substance use, and intimate partner violence (IPV) significantly increases Black women’s already high vulnerability to HIV infection, homelessness, and other severe adverse life experiences [6]. The rate of HIV diagnoses among Black women is 15 times as high as White women, and is among the leading causes of death among Black women ages 25–34 in the United States [7]. However, most evidence-based HIV prevention interventions have been developed and targeted towards White men who have sex with men. Few HIV prevention and intervention efforts have directly targeted vulnerable Black women who experience substance use issues, IPV, and other adverse life events.

**Fig 1:**
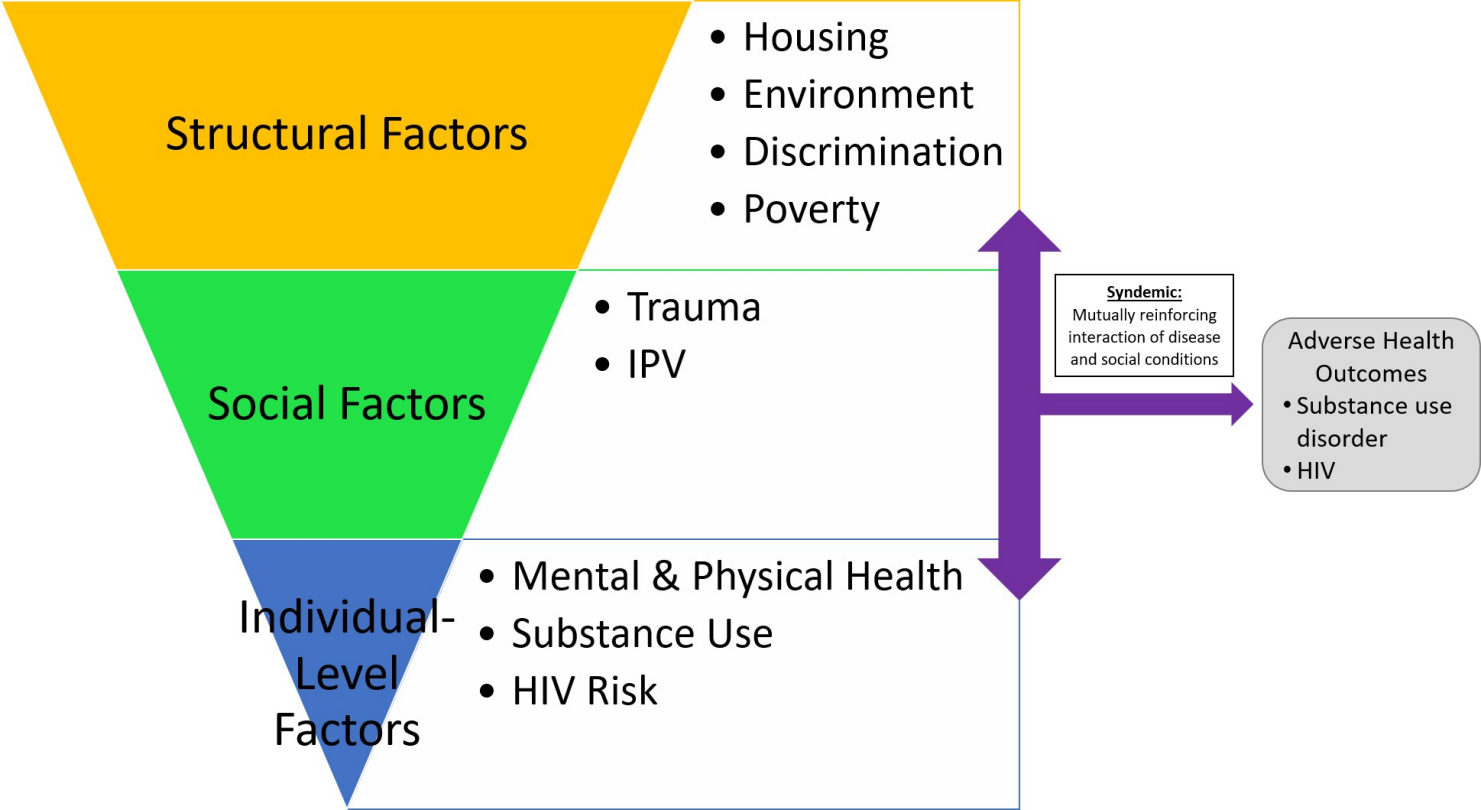
Representation of syndemic theory and factors from a socioecological perspective.

Syndemics theory posits that co-occurring adverse conditions interact synergistically within a population to heighten the negative effects of each other to contribute to increased disease burden should the conditions occur separately [8]. Additionally, syndemics theory uses a socioecological perspective that accounts for multiple levels of factors that affect health including structural, interpersonal, and individual factors [9] (Fig 1). Previous studies identified that housing instability and homelessness were associated with sexual risk behaviors [10–12] and sexual coercion [13].

Some individuals use substances to cope with structural factors [14,15]. For example, one study found that Black women who experienced more alcohol-related problems also reported more syndemic factors: they did not have health insurance, experienced childhood maltreatment, and lived in neighborhoods with high crime rates [16]. Studies have reported increased substance use among women who experience IPV [17,18], including a bi-directional relationship where women may use substances to cope with IPV or substance use impairs their ability to detect an escalating situation [19]. IPV also increases HIV risk as negotiating male condom use may be unsafe and lead to IPV [13,20]. Black women who use illicit drugs or exchange sex for money, drugs, shelter, or other goods are among the most vulnerable for HIV infection [21,22].

Black women who experience high levels of trauma including IPV, childhood sexual abuse (CSA), and other forms of violence have greater needs for substance use and mental health treatment yet have less access to care and when they do access care, it is of lower quality [23]. One study found that post-traumatic stress symptoms mediated the relationship between childhood trauma and problematic substance use, and women were significantly less likely to report and seek treatment for substance use [24]. Women are more likely to be caregivers and Black women are significantly more likely to be reported to child protective services compared to White women for the same rate of substance use [24], which may decrease seeking help for substance use concerns.

The application of syndemic theory on structural- and individual-level factors among Black women in the United States is understudied and urgently needed. More research is needed to understand structural- and individual-level factors that contribute to negative health outcomes among this population. Specifically, understanding experiences of syndemic factors in the context of substance use among highly vulnerable Black women will allow for the development of targeted interventions to address factors contributing to health disparities in this population, specifically regarding HIV prevention. It is essential to better understand the syndemic factors in the context of substance misuse and recovery among Black women at risk for substance use disorders and subsequent adverse consequences such as structural factors (i.e., housing, employment) and individual factors (i.e., IPV, HIV/STDs).

The present study aimed to qualitatively explore similarities and differences of syndemic factors in terms of substance use among Black women at high risk for HIV over a period of six months. While we know syndemic factors are mutually reinforcing and negatively impact Black women’s health, the present study adds to syndemic theory by enhancing understanding of substance use across multiple syndemic factors. Exploring substance use and misuse from a syndemic theory approach will inform the tailoring of interventions for Black women who misuse alcohol, marijuana, and other drugs.

## Materials and methods

The present study is part of a larger study exploring pre-exposure prophylaxis (PrEP) interest, and facilitators and barriers to PrEP adoption among Black women at high risk for HIV. The [Blinded for Review] Institutional Review Board approved all study procedures.

### Participants

We conducted semi-structured in-depth interviews with 31 Black women (age *M* = 32.13 years, range 18–57 years) four times over six months from July 2016 –April 2017. Purposive and snowball sampling recruitment occurred in Milwaukee, WI at various community events, a clinic, and by participant referrals. Participants were provided the option of handing out study cards to peers with the study information, eligibility criteria, and contact information. Participants were not compensated for referrals and interested women contacted the study; research team members never contacted potential participants.

To ensure participants were Black women at high-risk for HIV, we implemented a multi-step process to screen for eligibility. Initial eligibility criteria, which was determined prior to signing the informed consent, included being 18 years or older, identified as a cisgender woman and Black/African American, had unprotected vaginal or anal sex with a man in the past 30 days, were HIV-negative or of unknown status, and spoke fluent English. Eligibility screening occurred either over the phone or in-person. Then an in-person interview was set up where the study was further explained at length. If they chose to participate, they signed the informed consent form, and completed a contact sheet to include them in follow-up interviews. We applied additional eligibility criteria to ensure HIV risk after the participant signed the informed consent form, due to the length and sensitive nature of the additional questions. The additional criteria included having at least one of the following risk factors, although due to experiencing syndemic factors, most participants had more than one: experienced physical, sexual, or psychological IPV in the past 3 months (adapted from the Conflict Tactics Scale-2 [25]; engaged in sex exchange or survival sex in the past 3 months [26]; or engaged in problematic substance use in the past 30 days defined as any illicit drug (except for marijuana), 8+ drinks of alcohol per week or 4+ drinks on one occasion [27], or marijuana use 14+ times per month [28]. This portion of the eligibility criteria occurred via a structured, audio-recorded interview. Ineligible participants were compensated $10 for their time. Of the 31 participants in the present study, one did not complete the fourth interview and one only completed her first interview. One participant was removed from the study because she disclosed after two interviews that she was HIV-positive. Six participants were ineligible after completing the structured interview, and of five women who contacted the study or provided their phone number, three were unresponsive, one’s number was disconnected, and another continually rescheduled and eventually was unresponsive.

### Framework and data collection

We conducted a longitudinal, qualitative study in which eligible participants completed a semi-structured, in-depth, audio-recorded interview at baseline, one, three, and six months. Four interviews were conducted to explore changes in participants’ circumstances and experiences of syndemic factors. Syndemic theory, including common structural factors, guided the development of the interview guide to explore adverse life experiences. The baseline interview discussed topics such as their daily life, housing, income and employment, racial and gender identity, social support, resiliency, CSA, current and former relationships, IPV, substance use and misuse, number and types of sex partners, condom use, condom negotiation, sex exchange, and sex under the influence of drugs or alcohol. Sex exchange was defined to participants as having sex for money, drugs, money to buy drugs, food, shelter, or any other goods. The follow-up interviews did not cover the topics of CSA or racial and gender identity, but addressed the remaining topics and changes in each of those topics since the previous interview. Participants were contacted via text or phone call every two to three weeks to increase retention. Participants were compensated increasing amounts of $25, $30, $35, and $45 for their interviews.

Interviews were conducted in English by female interviewers. LAN, who was a post-doctoral fellow during the study, conducted over 90% of the interviews and the remaining interviews were conducted by a full-time research assistant who had prior experience conducting 50% of the qualitative interviews among gang-involved Black and Latina youth for an R01 grant, and was trained by LAN for this study. Participants were informed during the consenting process that the study was about ascertaining Black women’s knowledge and interest in PrEP. They were also informed that they would be asked personal and sensitive information, that they could skip any question, take a break, or stop the interview at any time. Participants had no relationship with LAN and some knew the research assistant. Thus, LAN conducted all interviews with participants who knew the research assistant.

A majority of interviews (~75%) took place in participants’ homes in which case a research assistant was present with the interviewer and participant. A research assistant who knew a participant was not present for any interview. Participants were told ahead of time to ensure there was a private place for the interview to be held where no one could listen. Participants who chose to travel to [first author’s] office with a designated interview room did not have a research assistant present and were reimbursed $5 for travel. Participants who arranged for childcare during their interviews were reimbursed $15, regardless of location, to ensure there was privacy. Interviews lasted between 30 minutes and 2.5 hours. Field notes were taken for context, environment, and unusual circumstances to supplement the audio recordings. At the end of each interview, participants were provided a comprehensive resource guide that included substance use treatment services, mental health services, housing and shelters, crisis intervention, family violence, food pantries, OB/GYN services, and HIV/STD testing sites. Data collection continued until saturation. Due to time limitations, we were unable to have participants review their transcripts.

### Substance use measures

For the present study, misuse or problematic use of substances was measured during the structured interview to determine eligibility using the following definitions: in the past 30 days, use of any illicit drug (except for marijuana); 8+ drinks of alcohol per week or 4+ drinks on one occasion [27]; or marijuana use 14+ per month [28]. Substance use was further explored in the semi-structured interviews to confirm responses from the structured interview and to determine context regarding substance use. Self-medicate was defined when the participant disclosed that they misused a substance in response to self-described mental health symptoms. Recovery was defined when a participant was no longer using a substance they misused in the past and actively sought to improve their health and wellness. Although they may have used or misused a different substance during the study, they were still deemed in “recovery” because they did not relapse to their previous drug of choice. We did not conduct a clinical interview to determine DSM-V diagnosis for a substance use disorder. Recovery was determined by participants disclosing that they misused or were addicted (their own words) to alcohol or drugs in their past for at least 3 months, and did not relapse by misusing the substance they self-described as misusing or were addicted to in their past.

### Data analysis

All interviews were transcribed verbatim and coded and analyzed in MAXQDA. Transcripts were analyzed using thematic content analysis [29] to systematically identify primary themes. These themes were developed into a preliminary codebook that were further refined over multiple iterations through team coding of additional baseline and follow-up interviews. The codebook was finalized once no new themes emerged and consensus was achieved among the first author and two research team members. For the present study, the first and second author re-analyzed data to explore the role of syndemic experiences within the context of problematic substance use. All interviews (n = 120) with a drug use, marijuana, or alcohol code were extracted and both authors analyzed each line systematically to identify primary themes regarding syndemic theory. Each participant’s interviews were analyzed sequentially to explore changes in substance use/misuse and experiences of syndemic factors. Participant ID numbers are used to protect participants’ privacy; their real ages are reported.

## Results

**Fig 2:**
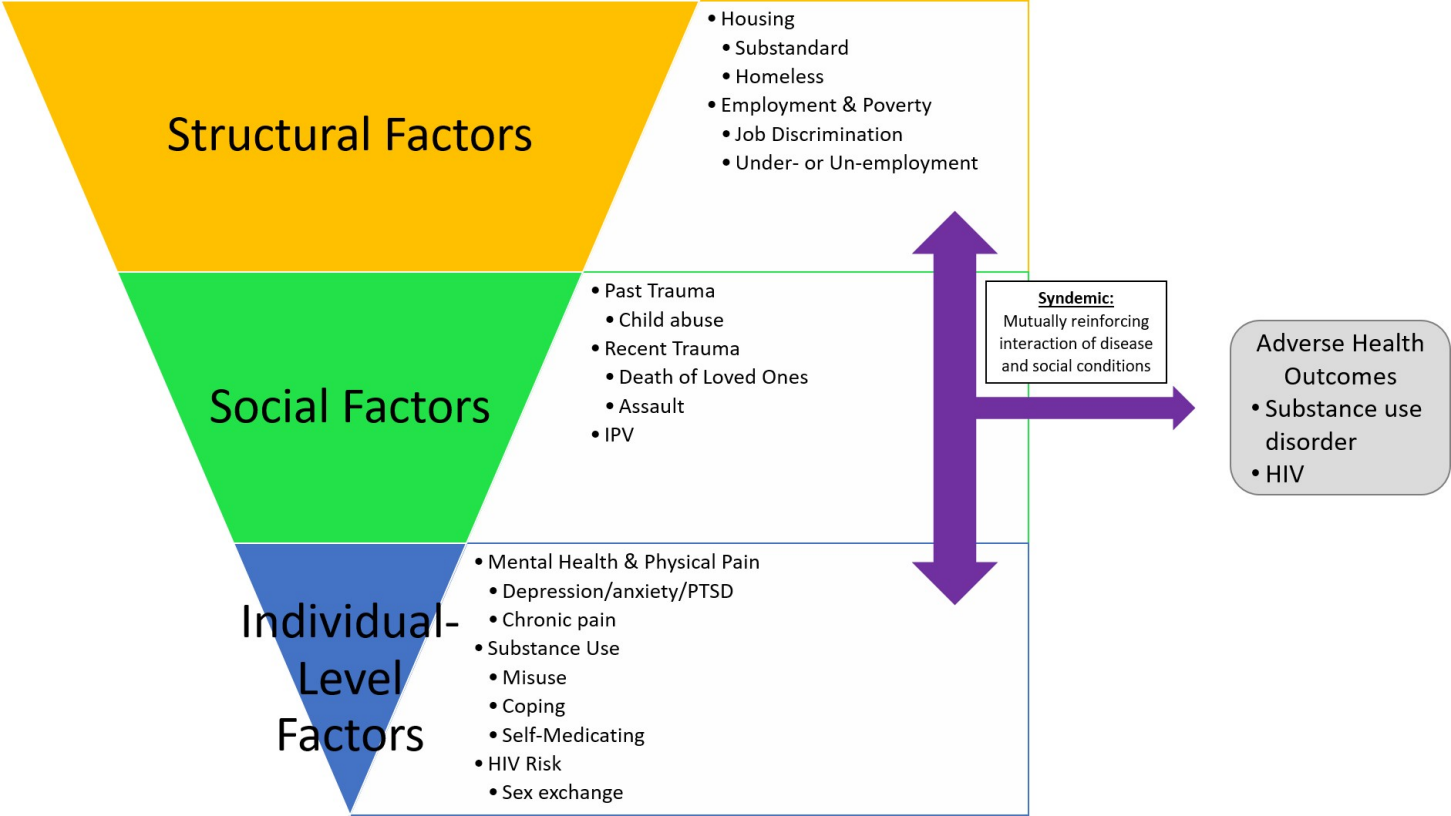
Characterizing syndemic factors among Black women at-risk for HIV infection.

Among all 31 participants, during the study 18 reported misuse of alcohol and 20 reported using alcohol as a negative coping strategy. In terms of marijuana, 17 reported misuse of marijuana and 23 reported using marijuana as a negative coping strategy. Six participants reported use of other illicit drugs. Almost all participants used alcohol, marijuana, and/or another illicit drug throughout the 6-month study period (Table 1). Two groups emerged during data analysis: 1) a “Recovery” Group which included participants who were in recovery from their drug of choice (n = 11, 7 of whom misused alcohol or marijuana during the study) and actively sought to improve their health and wellness, and 2) those who actively used substances during the study with no intent to change their substance use behavior (n = 20). Participants in the recovery group were characterized as abstinent from their previous drug of choice that created significant problems in their lives. All but one participant abstained from their prior substance of choice for over one year prior to the study and one quit using crack cocaine directly before the study. In the recovery group, some participants reported continued use of other substances such as alcohol, marijuana, and prescribed pain medications, but did not use their original drug of choice. The remaining recovery group participants reported being abstinent from all substances. Participants in the active use group did not abstain from any substance, currently misused alcohol/marijuana or another substance, or used alcohol/marijuana as a coping mechanism. Three major themes related to substance use emerged that aligned with syndemic theory: 1) structural factors, 2) adverse life events, and 3) individual-level factors (Fig 2). We explored each of these themes by substance use group.

**Table 1:** Participant characteristics and syndemic factors experienced by substance use group.

	Recovery Group (n = 11)					Active Use Group (n = 20)				
	T1	T2	T3	T4	Total	T1	T2^a^	T3^a^	T4^b^	Total
	n (%)	n (%)	n (%)	n (%)	n (%)	n (%)	n (%)	n (%)	n (%)	n (%)
Demographics										
Age (*M*, (*SD*))					38.00 (10.69)					28.70 (7.89)
Children (*M*, (*SD*))					3.91 (2.88)					2.10 (1.52)
Structural Factors										
Housing										
Substandard	8 (72.73)	7 (63.64)	7 (63.64)	6 (54.55)	8 (72.73)	11 (55.00)	9 (47.37)	8 (42.11)	10 (55.56)	13 (65.00)
Doubled Up/Overcrowded	1 (9.09)	2 (18.18)	1 (9.09)	1 (9.09)	2 (18.18)	8 (40.00)	8 (42.11)	7 (36.84)	8 (44.44)	10 (50.00)
Homeless	0 (0.00)	0 (0.00)	1 (9.09)	1 (9.09)	1 (9.09)	1 (5.00)	1 (5.26)	1 (5.26)	1 (5.56)	1 (5.00)
Unstable	0 (0.00)	2 (18.18)	1 (9.09)	1 (9.09)	3 (27.27)	4 (20.00)	4 (21.05)	8 (42.11)	5 (27.78)	9 (45.00)
Employment										
Part-Time	2 (18.18)	3 (27.27)	5 (45.45)	4 (36.36)	6 (54.55)	8 (40.00)	6 (31.58)	11 (57.89)	9 (50.00)	12 (60.00)
Full-Time	1 (9.09)	1 (9.09)	1 (9.09)	0 (0.00)	2 (18.18)	2 (10.00)	3 (15.79)	2 (10.53)	4 (22.22)	5 (25.00)
Odd jobs	2 (18.18)	3 (27.27)	1 (9.09)	2 (18.18)	3 (27.27)	7 (35.00)	9 (47.37)	7 (36.84)	6 (33.33)	11 (55.00)
Seeking (More) Employment	4 (36.36)	1 (9.09)	4 (36.36)	4 (36.36)	7 (63.64)	7 (35.00)	3 (15.79)	7 (36.84)	5 (27.78)	10 (50.00)
Adverse Life Experiences										
Past Trauma										
Childhood Sexual Abuse	--				7 (63.64)	--				7 (35.00)
Childhood Physical Abuse	--				5 (45.45)	--				7 (35.00)
Rape	-				5 (45.45)	--				2 (10.00)
Recent Trauma										
Assault	0 (0.00)	0 (0.00)	0 (0.00)	0 (0.00)	0 (0.00)	0 (0.00)	1 (5.26)	2 (10.53)	3 (16.67)	4 (20.00)
Assault of Loved One	0 (0.00)	0 (0.00)	0 (0.00)	1 (9.09)	1 (9.09)	1 (5.00)	0 (0.00)	0 (0.00)	0 (0.00)	1 (5.00)
Death of Loved One	1 (9.09)	0 (0.00)	1 (9.09)	0 (0.00)	1 (9.09)	1 (5.00)	3 (15.79)	1 (5.26)	1 (5.56)	4 (20.00)
Incarceration of Loved One	2 (18.18)	0 (0.00)	0 (0.00)	0 (0.00)	2 (18.18)	0 (0.00)	0 (0.00)	0 (0.00)	0 (0.00)	0 (0.00)
Substance Use^c^										
Previous Alcohol Misuse	--				5 (45.45)	--				1 (5.00)
Alcohol Misuse	4 (36.36)	5 (45.45)	2 (18.18)	2 (18.18)	5 (45.45)	9 (45.00)	5 (26.32)	4 (21.05)	5 (27.78)	13 (65.00)
Alcohol to Cope	6 (54.55)	2 (18.18)	2 (18.18)	2 (18.18)	6 (54.55)	9 (45.00)	6 (31.58)	3 (15.79)	5 (27.78)	14 (70.00)
Previous Marijuana Misuse	--				6 (54.55)					10 (50.00)
Marijuana Misuse	4 (36.36)	3 (27.27)	2 (18.18)	3 (27.27)	6 (54.55)	8 (40.00)	7 (36.84)	9 (47.37)	9 (50.00)	13 (65.00)
Marijuana to Cope	4 (36.36)	2 (18.18)	2 (18.18)	1 (9.09)	6 (54.55)	12 (60.00)	9 (47.37)	8 (42.11)	6 (33.33)	17 (85.00)
Previous Other Drug Misuse^d^	--				6 (54.55)	--				2 (10.00)
Other Drug Misuse^e^	4 (36.36)	1 (9.09)	1 (9.09)	1 (9.09)	4 (36.36)	2 (10.00)	1 (5.26)	1 (5.26)	1 (5.56)	2 (10.00)
Intimate Partner Violence (IPV)^c^										
Previous IPV	--				8 (72.73)	--				15 (75.00)
Physical IPV	1 (9.09)	1 (9.09)	2 (18.18)	2 (18.18)	4 (36.36)	10 (50.00)	2 (10.53)	3 (15.79)	0 (0.00)	12 (60.00)
Psychological IPV	8 (72.73)	4 (36.36)	6 (54.55)	7 (63.64)	11 (100.00)	13 (65.00)	7 (36.84)	8 (42.11)	6 (33.33)	18 (90.00)
Sexual IPV/Coercion	6 (54.55)	6 (54.55)	3 (27.27)	4 (36.36)	9 (81.82)	10 (50.00)	1 (5.26)	4 (21.05)	2 (11.11)	10 (50.00)
Sex Exchange^c^										
Previous Sex Exchange	--				6 (54.55)	--				1 (5.00)
Sex Exchange	4 (36.36)	2 (18.18)	0 (0.00)	0 (0.00)	4 (36.36)	4 (20.00)	0 (0.00)	1 (5.26)	1 (5.56)	4 (20.00)

T1 = baseline; T2 = 1-month; T3 = 3-month; and T4 = 6-month interviews.

^a^n = 19 due to attrition.

^b^n = 18 due to attrition.

^c^Previous denotes use prior to the study for at least 3 months.

^d^Other includes: crack cocaine, cocaine, anxiety/antidepressant medication, combination marijuana and PCP.

^e^Other includes: crack cocaine, pain medication, cocaine, K2 (synthetic marijuana). Substance misuse: Alcohol– 8+ drinks/week or 4+ drinks/occasion; Marijuana misuse–smoke 14+ days/30 days; Other drugs–any illicit/other drug in past 30 days. Substance to cope: using a substance as a coping strategy in response to a negative situation or feeling, not necessarily misuse.

### Structural-level factors

Structural violence, such as lack of access to housing, affordable housing, homelessness, lack of access to employment or employment without adequate pay, and discrimination led to substance use among many participants across both groups.

#### Housing

Most participants were displaced from their housing, lived in substandard housing and/or were homeless (Table 1). Substandard housing included participants whose residences had physical problems that were frequently ignored by property owners (e.g., pest infestation, non-working plumbing, lack of appliances, holes in walls or floors, leaks, etc.). Both the recovery and active use groups reported using alcohol or marijuana to cope with substandard or unstable housing situations. The active use group reported more daily substance misuse (Table 1) and adverse consequences such as getting drunk, passing out, or difficulty paying bills due to substance use. For example, participant 135 (35 years old) increased her alcohol consumption by her second interview, and was drinking daily to cope with being displaced and the difficulty of finding affordable housing. Eventually, participant 135 found an apartment and doubled up with her sister and sister’s family because she could not afford housing for her and her three children on her own. Alternatively, the recovery group reported lower quantities and frequency of substance use (Table 1). Participant 107 (34 years old) was displaced from her apartment because of the extremely poor living conditions that the landlord refused to fix and she smoked marijuana once to cope. Unfortunately, participant 107 could not find affordable housing and moved into a shelter for the remainder of the study (Table 2, Structural Factors: Housing).

**Table 2:** Syndemic factors experienced among recovery and active use groups.

Syndemic Theory	Group Differences	Group Similarities	Representative Quote: Recovery Group	Representative Quote: Active Use Group
**Structural Factors**				
Housing	**Recovery Group** participants reported lower quantities and frequency of alcohol/marijuana use due to housing concerns; **Active use group** reported daily use and more consequences from substance use, such as getting drunk, passing out, difficulty paying bills due to substance use.	Both groups reported alcohol or marijuana use to cope with housing instability	(107, 34 y.o., T2): “I did puff some marijuana though. Before I moved out of the place, I had got the power turned off, but I didn’t think they were gonna turn the power off right away. So it was like a couple days I had to spend in the dark. I had to cope some type of way, so I did. I smoked a little bit…it’s gonna be the only way I’m gonna be able to sleep.” *Outcome*: *Unable to find affordable housing; moved into a shelter*	(135, 35 y.o., T2): “I drunk a little more this week. I felt a little more stressed. . .I probably drinked about the whole week…We [sister] tryin’ to put the pieces together, we go get red books and sit down and we circle the ones that we can afford.” *Outcome*: *Found an apartment and doubled up with sister and her family because Participant 135 could not afford housing on her own*.
Employment	**Recovery group** reported stress caused by racial and/or gender discrimination at place of employment. **Active use group** reported more challenges with finding and maintaining employment.	Both groups reported alcohol and marijuana use to cope with employment-related stress	(123, 30 y.o., T1): “I was the only woman in my job and also the only African-American…It was a nightmare…Every day that is why the wine came in.” *Outcome*: *Injured on the job and fired because she could no longer work*. *She did not know how to file for unemployment and was unaware that it is illegal to be fired for an injury that occurred at the workplace*.	(143, 26. T4): “[I tend to use substances] when I just need to take a break from everything now…I get stressed out a lot…I just been applying for all different jobs. Just ready for something to come through so I can really start adding extra money to the money I have saved so I can just [move out of living situation].”
**Adverse Life Experiences**				
Past Trauma	**Recovery group** reported prior substance use, particularly illicit substances, in response to past traumas; **Active use group** reported less past traumas and less use of substances, particularly illicit substances, to cope with trauma	Both groups reported substance use after experiencing traumas in the past	(141, 46 y.o., T1): “I was molested. My first experience I was maybe 6 years old…maybe about 12 when [my stepfather] penetrated me and he stopped when I was 13 ‘cause I started runnin’ away from home…so after that I started havin’ sex on my own. And I was drinkin’, smokin’ weed, I tried happy sticks, all kind of stuff [to cope].” *Outcome*: *Did not use any illicit drugs throughout the study and drank alcohol socially*.	(140, 21 y.o., T1): “I started smoking [marijuana] to irritate my mama, ‘cause she don’t like the fact that I smoke. I smoke because it’s a lot of stuff that don’t have to deal with, and a lot of people tell me, they were smokin’ before I didn’t smoked, they was tellin’ me that they would smoke to help them deal with problems in life. Now that’s what I feel like it help me do.” *Outcome*: *Did not misuse marijuana directly after traumas (CSA and rape); misused alcohol and marijuana for the first half of the study*.
Recent Trauma	**Recovery group** did not use drug of choice to cope w/ recent traumas, but reported alcohol and marijuana use to cope; **Active use group** reported more substance use to cope w/ recent traumas.	Both groups resorted to alcohol and marijuana use after recent traumas	(130, 47 y.o., T1): “I just refuse to put myself through [using crack again]. Because of the emotional things I’ve been through as of late, I wouldn’t dare trust myself because sometimes I have days were I just feel like man, fuck it [I’m going to use again]—but then I think I just have too much [to lose].” *Outcome*: *Never relapsed during study but did use alcohol and marijuana to cope with recent traumas*.	(105, 30 y.o., T4): “Somebody get on my nerves or make me a little depressed to the point sometime I just tryin’ to drink just to go to sleep…[I’m depressed about] everything that been goin’ on basically, jumpin’, stabbing.”
**Individual-Level Factors**				
Coping Strategies	**Recovery group** reported less substance use in response to stress and more for coping with physical pain; **Active use group** reported more substance use in response to stress.		(130, 47 y.o., T1): “I smoke marijuana for my pain. I like the high, but because I don’t get any narcotics or anything, I smoke weed for my pain. Sometimes smoking weed is the only way I can walk some days.” *Outcome*: *She suspected she had multiple sclerosis but could not find adequate healthcare and continued to use marijuana throughout the study to cope with pain*.	(137, 37 y.o., T4): “I spent $80 since we’ve been off work since Friday on weed. I think I’m stressing out…[my children getting on my nerves, [roommate] is getting on my nerves, her daughter get on my nerves…like I be lonely—it’s a lot of stuff. My son just had surgery, so I got a lot of stuff on me.”
Substance Use	**Recovery group** reported overcoming urges to use their drug of choice. **Active use group** reported more substance use and had more difficulty refraining from substance use.	Both groups resorted to substance use when faced with stressors	(119, 34 y.o., T2): “Sometimes when I get really stressed out or nervous I do want to resort back to takin’ [anxiety and depression] pills, but I haven’t…’cause that was sorta a problem for me in the past. I would just go overboard, but it never really helped me, it just turned into a addiction.” *Outcome*: *Never relapsed with pills or marijuana*, *only drank socially*.	(116, 36 y.o., T1): When I was drinking, I was a monster I guess. Like my kids, they would run away from me, they didn’t like being my family. They argued, I always fought with them and I always hung out in the streets, partying and doing wrong…It’s been about 6 months [I’ve been sober]. (116, 36 y.o., T2): “[I had a drink] two weeks ago. Just half a can…I was stressed out with the fighting and arguing.” (116, 36 y.o., T3): “If that was normally right now I would drink from the time I wake up to the time that I go to sleep, but now I just drink one bottle, half a pint…I don’t do it every day. Like every other week…I don’t really like drinkin’ like that but I guess I start drinkin’ and doing’ that when I get mad at somebody.” *Outcome*: *Drank alcohol and smoked marijuana daily by her last interview*.
Self-Medicating for Mental Health Symptoms	**Recovery group** reported less use of substances in response to mental health symptoms; **Active use group** reported using substances more frequently to self-medicate due to mental health systems.	Both groups reported using alcohol and marijuana to cope with mental health symptoms	(130, 47 y.o., T3): “I wish I could have smoked every day because I was just a wreck. I was having panic attack after panic attack…[but I didn’t smoke] because of money and morality because I’m trying to get my life right with God and I know drugs aren’t a part of God’s plan.” *Outcome*: *Continued to use marijuana throughout the study but frequency decreased*.	(136, 18 y.o., T1): “I smoke. That’s my [emotional] pain relief right there…I just feel like [the medication] don’t do nothing. I still have my mood swings, I still be happy one minute, sad one minute, mad one minute. It don’t do nothing to me so I deal with that through my weed.” *Outcome*: *Misused marijuana throughout most of the study and by her last interview only misused alcohol*.
Intimate Partner Violence	**Recovery group** reported more IPV but were less likely to cope with substance use—they did in the past but not anymore; **Active use group** reported less IPV but more substance use in response to IPV	Both groups reported history of substance use substances to cope with IPV during their lifetime	Many participants stated “I did in the past” referring to substance use in response to IPV. *Outcomes*: *None relapsed or increased substance use in response to IPV*.	(138, 22 y.o., T1): “I like the numb feeling…something to take [violent ex-partner] off my mind, which drinkin’ does.” *Outcome*: *Alternated between excessive alcohol and marijuana use*.
Sex Exchange	**Recovery group** reported engaging in sex exchange both in the past and during the study, often involved illicit drugs in the past, but not currently; **Active use group** reported less sex exchange and was usually survival sex.	Both groups engaged in survival sex	(130, 47 y.o., T1): “When I was out there prostituting, I didn't always have protected sex and I have [not] been tested in a while, but as it stands now, I dodged a bullet… When I used to be out there on them drugs and stuff, I compromised my integrity so much.” *Outcome*: *Engaged in survival sex early in the study*.	(101, 26 y.o., T1): “I lie, I did [exchange sex] one time to get some food in the house…just recently.” *Outcome*: *Used a condom during sex exchange; did not engage in survival sex during the study but engaged in other risk behaviors such as inconsistent condom use and multiple sex partners*.

#### Employment

Some participants used alcohol/marijuana to cope with being unable to find employment, whereas others used substances because of a difficult work environment or inadequate pay. The active use group reported more challenges with finding and maintaining employment (Table 1), such as participant 143 (26 years old). She struggled throughout the study with maintaining employment and finding an adequate-paying job, which led to doubling up (i.e., living with someone in an overcrowded housing situation) with friends in her second and third study interviews. Her alcohol and marijuana use increased by the end of the study to cope with unemployment and not having enough money to move into her own housing with her daughter. Participants in the recovery group reported their financial stress because of racial and/or sex discrimination that led to undesirable working environments and difficulty finding employment. One participant, 123 (39 years old), reported experiencing discrimination at her job both because of her race and sex. Unfortunately, she was injured on the job and she was fired because she could no longer work. She did not know how to file for unemployment and was unaware it was illegal to be fired for an injury that occurred at the workplace (Table 2, Structural Factors: Employment).

### Adverse life events

Many participants experienced adverse life events in their past or during the study, and often used substances to cope with the traumas.

#### Past trauma

A majority of participants were physically (n = 3), sexually (n = 8), or both physically and sexually (n = 5) abused during their childhood, and several were previously raped (n = 7; Table 1). These traumas often led to being removed from their home or running away. Participants in the recovery group reported more past traumas and often recognized that these traumatic experiences led to their initiation of substance use, particularly illicit drug use, at a young age. Participant 141 (46 years old) was molested and sexually abused by her stepfather, which led her to run away as a teenager and use substances to cope. Active use participants reported fewer traumas in their past and tended to not initiate substance use as a direct result of past traumas (Table 1). For example, participant 140 (21 years old) did experience both childhood sexual and physical abuse, but began smoking marijuana later. She expressed that she initially started smoking marijuana to irritate her mother and because friends who smoked told her it helped them cope with their problems (Table 2, Adverse Life Events: Past Trauma).

#### Recent trauma

Many participants experienced traumas during the study such as assault, the assault or death of a loved one, and family or significant others who were incarcerated. The active use group reported more traumas during the study (Table 1) and used alcohol/marijuana to cope. Participant 105 (30 years old) was physically assaulted twice during the study, including being stabbed, and reported drinking alcohol to cope with her traumatic experiences. Participants in the recovery group who experienced adverse life events during the study reported cravings and urges to use their drug of choice but none relapsed during the study. For example, participant 130 (47 years old) reported wanting to use crack cocaine again to cope with recent adverse life experiences but did not because she had too much to lose going back to that lifestyle. Prior to the study, participant 130 gave birth and was forced to give her child up for adoption because she relapsed with crack cocaine. She did not want to lose her youngest child because of relapsing again so, while she experienced significant urges to use, she continued her sobriety from crack cocaine throughout the study (Table 2, Adverse Life Events: Past Trauma).

### Individual-level factors

Participants used substances for coping strategies with stress and physical pain relief, to self-medicate to cope with mental health symptoms, to cope with IPV, and engaged in sex exchange.

#### Coping strategies

Several participants used alcohol/marijuana to help relieve general stress and relax, or to manage physical pain. Active use participants reported more substance use in response to stress (Table 1), whereas recovery group participants reported less substance use for stress and more for pain management. Participant 137 (37 years old) began smoking marijuana only on weekends halfway through the study and by her last interview she reported frequent marijuana misuse to cope with life stressors. Yet, participant 130 (47 years old), reported smoking marijuana to help cope with chronic pain (Table 2, Individual Factors: Coping Strategies).

#### Substance use

While most participants engaged in substance use during the study, the active use group reported more frequent substance use (Table 1) and difficulty refraining from substance use. For example, participant 116 (36 years old) was sober when the study started but by her second interview, drank alcohol because she was stressed; by her third interview she drank alcohol and smoked marijuana more frequently, and by her last interview she drank and smoked daily and began using in the morning. The recovery group tended to use substances less frequently and overcame temptation to relapse. Participant 119 (34 years old) was previously dependent on anxiety and depression medication, and misused marijuana in the past. When she was stressed or nervous she was tempted to use again, but she never did with pills or marijuana and reported only drinking alcohol socially (Table 2, Individual Factors: Substance Use).

#### Self-medicating for mental health symptoms

Several participants would self-medicate with alcohol/marijuana to cope with mental health concerns. Many participants used alcohol or marijuana instead of seeking counseling or taking medication for their mental health symptoms because of lack of awareness, high cost, lack of transportation, or because they felt that counseling and/or medication was not effective. This was more frequent among the active use group as opposed to the recovery group. For example, participant 136 (18 years old) explained that she thought the medication for her diagnosed bipolar disorder did not work and instead smoked marijuana. Yet participant 130 (47 years old) experienced panic attacks and was tempted to smoke marijuana every day but did not because she could not afford to and she felt smoking marijuana daily did not align with improving her life (Table 2, Individual Factors: Self-Medicating for Mental Health Symptoms).

#### IPV

All participants experienced some form of IPV either in their past or during the study and many used substances to cope with IPV (Table 1). The active use group reported fewer instances of IPV but more substance use to cope with IPV. The recovery group reported more instances of IPV both currently and in their past. This group reported using substances to cope in their past but used them less frequently in response to experiences of IPV during the study. Participant 138 (22 years old) experienced severe physical IPV that occurred directly before her first interview and reported that she misused alcohol to forget about her ex-boyfriend. Throughout the study, she continued to misuse alcohol, then quit drinking and misused marijuana, and by her last interview she was drinking again but not as excessively compared to the beginning of the study. When participants in the recovery group were asked about substance use in response to IPV, most reported “I did in the past” but no longer used substances to cope with recent IPV (Table 2, Individual Factors: Intimate Partner Violence).

#### Sex exchange

Seven participants engaged in sex exchange in their past and eight participants engaged in sex exchange throughout the study (Table 1); two had sex for money to buy drugs and five engaged in survival sex (participants not mutually exclusive). Survival sex was defined as exchanging sex for money to pay for bills or buy food, for food, for shelter, or any other goods to improve their circumstances. Participants in the recovery group reported illicit substance use more frequently when they exchanged sex in the past or during the study. For example, participant 130 (47 years old), discussed when she exchanged sex in her past, she used crack cocaine frequently and used condoms inconsistently. She recognized that she was lucky to not have contracted HIV or another sexually transmitted infection and expressed that “I compromised my integrity so much.” Participants in the active use group were less likely to exchange sex and when they did, it was more often survival sex. Participant 101 (26 years old) exchanged sex and used a condom a month before her first interview for money to buy food for her and her three children. Although she did not exchange sex during the study, she engaged in other risky sexual behaviors such as inconsistent condom use and had multiple sex partners (Table 2, Individual Factors: Sex Exchange).

## Discussion

The present longitudinal, qualitative study analyzed qualitative interviews from 31 vulnerable Black women to better understand their substance use in the context of syndemic factors such as structural factors, adverse life events, and individual factors and how each group (recovery versus active use) reported experiences differently. This is a unique addition to the literature as very little longitudinal research has been conducted examining substance misuse in terms of syndemic factors among this highly vulnerable, difficult-to-retain population.

Two groups emerged in the data: recovery group and active use group. By using a longitudinal design, we were able to observe changes in substance use among participants throughout the course of the study. Several participants changed their substance use patterns during the study, which allowed us to identify these two distinct substance use groups. After considering substance use through the lens of syndemic theory, we identified specific themes regarding syndemic factors and substance use changes over time.

Results support other studies that demonstrated the concomitant nature of structural factors and substance use [14–16]. Structural factors appeared to increase substance misuse in response to substandard housing, housing instability, displacement, homelessness, lack of employment, underemployment, or racial/gender discrimination. Participants in the recovery group reported less substance use than the active use group, but reported increased stress resulting from discrimination compared to the active use group. This may be a product of the numbing properties of psychoactive drugs resulting in heightened stress response to similar environmental cues among people in recovery. Of note, most participants in the recovery group did not receive or complete evidence-based substance use treatment such as coping skills for stress management and trauma. Recovery group participants continued to use alcohol and marijuana as a strategy to cope with structural challenges. Lack of access to affordable stress management skills programs such as counseling and yoga may contribute to self-medicating with substance use. This highlights resiliency among this population in their recovery accomplishments with minimal formal treatment and recovery supports; however, increasing access to evidence-based treatment and recovery supports may reduce stress, alcohol and marijuana use, and improve mental health functioning to aid in long-term recovery. Our data also highlighted an important need to provide vulnerable Black women with education and support related to workers’ compensation and unemployment benefits, as well as affordable legal representation. Programs to increase affordable and stable housing, particularly with case management to assist with crises and consultation, are essential [30].

In line with studies demonstrating associations between CSA and substance use [31,32], results demonstrated mutually reinforcing syndemic factors of CSA and other adverse life events, and substance use, particularly over time. Participants in the recovery group who discussed adverse life experiences such as childhood sexual and physical abuse, sexual assault, and initiation of substance use because of trauma were less likely to use substances later in the study to cope with traumas compared to the active use group who used substances more frequently in response to traumas. This longitudinal study reflects better insight into the etiology and triggers related to their past and current substance use. Further, the recovery group reported coping with urges and cravings through a focus on self-improvement and maintaining a higher moral standard. They had a desire to avoid consequences they had previously experienced as a result of illicit drug use. Patterns of current substance use among recovery group participants demonstrate the utility of a harm reduction approach to decreasing risky substance use among vulnerable Black women. Further, these data suggest that focusing substance use treatments within the individual’s moral framework and focusing on life purpose, goals, and avoiding negative consequences may be important treatment adaptations for this population.

Participants across both groups reported individual factors that led to increased substance use, often related to coping with significant life stressors. This behavior was more common among the active use group as participants in the recovery group used substances less frequently. Participants used substances to cope with stressful life events, physical pain, to self-medicate mental health symptoms, and IPV [17,19,31,33–36]. Those in the recovery group used substances less frequently and were able to overcome cravings and urges, which prevented relapse with their primary drug; however, many participants in the recovery group continued to use unhealthy amounts of alcohol or marijuana. Participants may have experienced the Superwoman role where Black women tend to internalize stress at the expense of self-care [37]. Internalizing stress may play a role in self-medicating for mental health symptoms with substances. Alcohol and drugs provide an immediate and effective short-term method for coping; however, as demonstrated throughout the longitudinal aspects of the study, unhealthy use of substances often exacerbated consequences. By not prioritizing self-care and seeking mental health care, participants may have increased internalized stress in which they used substances to cope. Using substances to cope with stress, mental health and physical symptoms indicates the increased need for system interventions aimed at increasing access to appropriate treatment for this vulnerable population.

A majority of participants experienced IPV and some used substances in response to the abuse, or substance use led to the IPV, and others used substances to cope with sexual coercion during sex they experienced with their partners. While participants in the recovery group experienced more IPV in their past and during the study, during the study they used substances less frequently to cope with IPV compared with the active use group. Due to the overlapping nature of IPV and substance use [8], particularly among this population, future interventions should consider addressing IPV during substance use treatment.

Some participants exchanged sex, more often survival sex, particularly prior to the study and at baseline. Participants in the recovery group were more likely to have used illicit substances when they exchanged sex in their past. This again highlights the need for structural interventions to assist Black women in finding housing and financial stability [30] to avoid the need to engage in survival sex. Further, individual-level HIV prevention interventions for this population should address the syndemic effects associated with IPV, substance use, and HIV risk behaviors, warranting multi-level interventions instead of brief, singular-focused interventions.

The present study identified three themes consistent with syndemic theory from the context of substance use: 1) structural factors (i.e., housing and employment), 2) adverse life events (i.e., childhood physical and sexual abuse, sexual assault, assault), and 3) individual-level factors (i.e., substance use, IPV, sexual risk, physical or mental health). By identifying two substance use groups, recovery and active use groups, results demonstrated the reinforcing nature, and similarities and differences across syndemic factors. For example, most participants experienced housing and financial difficulties, and many participants used substances to cope. Financial difficulties also led participants to engage in transactional sex; those in the recovery group were more likely to engage in sex exchange for drugs or money to buy drugs, whereas the active use group tended to engage in survival sex. Additionally, many participants experienced adverse life events and increased substance use, or experienced mental health symptoms and self-medicated with substances. By examining the data from the context of substance use, we identified different ways in which substance use enhanced and/or resulted from structural-level factors, adverse life events, and individual-level factors. There are implications for future programs for Black women at risk for HIV to view substance use through the lens of syndemic theory and identify potential nuances based on current substance use (i.e., recovery vs. active use).

### Limitations

While this study contributes to the literature by presenting experiences of syndemic factors from the perspective of substance use and misuse among highly vulnerable Black women, it is not without its limitations. This study took place in Milwaukee among a small sample of Black women and therefore cannot be generalized outside of this geographic area or population. Due to the sensitive and illegal nature of many of the questions, participants may have underreported traumatic or risky behaviors such as initiating illicit substance use after childhood sexual abuse or frequency of illicit substance use. Future studies should expand this qualitative research using quantitative methods in addition to in-depth qualitative interviews, as well as collect hair samples or urine toxicology screens, which may provide more objective means of data collection related to recent drug use.

### Future research

The present data can be used to develop a future multi-level intervention addressing syndemic factors associated with substance use, structural challenges, and sex risk due to the limited interventions adapted specifically for vulnerable Black women at-risk for HIV infection. Future research should consider the role of substance use and cultural factors [38] among highly vulnerable Black women. Substance use interventions should consider including structural factors, such as how to search for housing and apply for low-income housing, and job training; social factors such as how to detect and leave relationships involving IPV and file restraining order; and individual factors involving mental health and safe sexual practices. Future studies should consider using a trauma-informed approach. One study implemented a brief, trauma-informed intervention to reduce IPV and mental health symptoms [39]. Using this strengths-based approach should be tailored for Black women to support those in recovery from substance use and for those actively using substances. A group-based approach should also be considered. A study conducted among Latina women implemented a brief group-level intervention that reduced HIV risk behaviors, exposure to IPV, and mental health symptoms [40]. Tailoring this type of group-level intervention for Black women by their substance use group may decrease negative outcomes. In addition, adding a housing component such as case management to increase permanent housing is essential [30].

## Conclusions

Highly vulnerable Black women are at high risk for unhealthy substance use and dependence. They often use substances in response to one or a combination of extremely stressful life events including structural factors (i.e., housing instability and under- or unemployment), previous and recent traumas, and individual-level factors such as self-medicating for mental health symptoms, coping with current or previous IPV, or having sex for money or drugs. Although engagement in substance use differed and was less frequent among participants in recovery from prior substance misuse, strategies to support relapse prevention and decrease the use of other substances is pertinent. Due to the extreme negative health consequences associated with substance use among vulnerable Black women, substance use interventions using a trauma-informed approach and support groups targeted for this population must be prioritized and include other information and skills (e.g., HIV and IPV risk reduction) in order to be effective.
